# Regulation of immune tolerance in hepatocellular carcinoma by liver diseases: a review

**DOI:** 10.1186/s13027-026-00764-5

**Published:** 2026-04-28

**Authors:** Jinyi Li, Yuanda Liu

**Affiliations:** 1 College of Traditional Chinese Medicine, Changchun University of Chinese Medicine, Changchun, China; 2 Department of Endoscopy Center, China-Japan Union Hospital of Jilin University, Changchun, China

**Keywords:** Hepatocellular carcinoma, Immune tolerance, Metabolic dysfunction associated steatohepatitis, Viral hepatitis, Alcoholic liver disease

## Abstract

The clinical benefit of immunotherapy for hepatocellular carcinoma (HCC) is often limited by innate immune tolerance. Traditional research focuses on the intrinsic escape mechanism of tumor cells or the generalized immunosuppression of the tumor microenvironment; however, it often ignores the chronic liver disease background of HCC patients with different etiologies. The aim of this review was to systematically discuss the mechanisms by which different underlying liver diseases, particularly metabolic dysfunction associated steatohepatitis, viral hepatitis (hepatitis B virus/hepatitis C virus) and alcoholic liver disease, specifically shape the liver immune microenvironment, thereby driving HCC to develop tolerance to immune checkpoint inhibitors. We further explored the molecular mechanisms by which various etiologies promote T cell exhaustion, expand immunosuppressive cells, and change the function of the gut-liver axis through unique pathways, such as metabolic dysfunction-associated steatohepatitis-related metabolic disorders and dysbiosis, sustained antigen stimulation and epigenetic regulation of viral hepatitis, and the hyperinflammatory chemokine network of alcoholic liver disease. In addition, this article systematically evaluates the potential synergistic or antagonistic effects of drugs used to treat these underlying liver diseases (e.g., lipid-lowering drugs, liver protective drugs, antiviral drugs, antifibrotic drugs, and new metabolic targeted drugs) on HCC immunotherapy. The objectives are to provide key mechanistic insights and strategic references for clinical combination drugs, as well as emphasize the corresponding individualized immunotherapy strategies for HCC caused by different diseases.

## Introduction

Hepatocellular carcinoma (HCC) is the sixth- and third-leading malignant tumor in terms of incidence and mortality rates, respectively, worldwide [1]. In recent years, the incidence of HCC has increased annually, posing a major threat to human health [2]. Due to the irreplaceability of liver function, surgical treatment of HCC is often characterized by numerous limitations. At present, the 5-year survival rate of patients with early and advanced HCC is 70% and < 10%, respectively; however, only 20% of patients are diagnosed in the early stage of disease [3]. Although immunotherapy provides a new option to prolong survival for patients with advanced HCC and recurrence, the overall response rate is only 15%–30% [4]. Immune tolerance greatly reduces the effect of immunotherapy. Therefore, in-depth understanding of the immune tolerance mechanism of HCC has clinical significance.

The tolerance of tumor immunotherapy is regulated by many factors, including immune escape factors such as the expression of programmed cell death 1 ligand 1 (PD-L1) by tumor cells themselves, the depletion of immune cells in the tumor microenvironment, the increase of immunosuppressive cells, the change of matrix structure, and the change of metabolic function [5, 6].The liver is at the core of systemic metabolism and has complex associations with the progression of tumors (extrahepatic tumors) [7]. Long-term chronic inflammation induced by tumorigenesis is first reflected in the change of liver function [8]. The liver can produce bile and regulate gut microbes [9]. Therefore, the gut-liver axis also plays an important regulatory role in the formation of HCC immune tolerance [10]. Current research indicates immune resesitance of hepatocellular carcinoma mainly involves three mechanisms: (1) dysfunction of immune cells (such as T cell exhaustion, unresponsiveness, and senescence [11, 12], (2) expansion of immunosuppressive immune cells (such as Tregs, MDSCs, and M2 macrophages [13, 14], and (3) overexpression of tumor immune checkpoint molecules (such as PD-1/PD-L1 and CTLA-4) [15]. In addition, the remodeling of stromal components such as fibroblasts in the tumor microenvironment is also a trigger for immune resistance [16]. Because of the functional importance of the liver, the immune tolerance of HCC is also tissue-specific. Although current research has elucidated many mechanisms of immunosuppression in HCC, there is a lack of effective interventions.

The occurrence of HCC is often accompanied by primary liver diseases, including metabolic dysfunction-associated steatohepatitis (MASH), viral hepatitis (hepatitis B virus/hepatitis C virus (HBV/HCV)) and alcoholic liver disease (ALD), etc., which eventually lead to cirrhosis and further progress to HCC [17]. In recent years, metabolic and inflammatory diseases have emerged as the greatest risk factors for the development of tumors. Therefore, exploring hepatocellular carcinoma in the context of MASH and viral infection holds significant clinical importance [18]. Only 13% of patients develop HCC without cirrhosis, but often with other liver diseases. Approximately 2% of patients are diagnosed with idiopathic HCC in the state of healthy liver [19]. However, the impact of primary liver disease on HCC has often been ignored in previous studies. There are similarities and differences in the immune microenvironment of HCC patients with or without cirrhosis induced by different primary diseases [20]. Therefore, exploring the mechanism of HCC immune tolerance based on liver underlying diseases may help us better understand the differences in immune tolerance from different HCC sources [21]. Based on the classification of liver basic diseases, the evidence can provide more precise immune tolerance solutions for patients with HCC. Therefore, in this review, we summarized the traditional regulation of HCC immunotherapy tolerance based on the changes of tumor cells, immune cells, and intestinal flora, as well as discussed the role of liver metabolic changes and organic changes in HCC immune tolerance.

## Role of MASH in immune tolerance of HCC

MASH, previously known as nonalcoholic steatohepatitis, is a risk factor for HCC. The incidence of MASH has been increasing annually, and approximately 20% of patients with HCC have this disease. MASH develops from metabolic dysfunction-associated steatohepatitis liver disease, previously known as nonalcoholic fatty liver disease. Currently, approximately 1.24 billion individuals worldwide suffer from metabolic dysfunction-associated steatohepatitis liver disease [22]. MASH can directly induce HCC with (or without) going through the process of cirrhosis [23], and its tumor microenvironment is unique compared to that of HCC caused by other etiologies. A meta-analysis based on 7,266 individuals reported that patients with MASH-HCC have a longer overall survival after undergoing HCC surgery compared to those with HCC of other etiologies [24]. However, in 2021, a study revealed that HCC patients with MASH did not respond to targeted therapy with PD-L1, indicating that those patients are more prone to immune tolerance [25]. Proteomic studies have also shown significant T-cell depletion and reduced numbers of cytotoxic T cells in MASH-associated HCC tissues [26]. This suggests that patients with MASH-HCC that has progressed to an inoperable stage and requires immunotherapy often do not receive effective treatment. Therefore, we have summarized current research progress on immune tolerance in MASH-related HCC. To enhance the reliability of the literature, we only included clinical studies and studies based on MASH-induced mouse models of primary HCC.

### MASH reshapes the HCC immune microenvironment

Insulin resistance and the excessive activation of the phosphatidylinositol 3 kinase/protein kinase B/mechanistic target of rapamycin (PI3K/AKT/mTOR) pathway are core manifestations of MASH. Insulin resistance exacerbates MASH, while the PI3K/AKT/mTOR pathway shapes the immune tolerance microenvironment through chronic inflammation and other mechanisms [27]. Macrophages play a crucial role in the progression of MASH and the formation of immune tolerance. During the MASH process, liver macrophages exhibit a phenotype similar to that of tumor-associated macrophages, accompanied by T-cell depletion [28]. In a MASH-HCC model, myeloid differentiation primary response 88 (MYD88) signaling in myofibroblasts enhances the secretion of C–C motif chemokine ligand 9 (CCL9), thereby promoting M2 polarization of macrophages and forming an immunosuppressive microenvironment [29]. RNA N^6^-methyladenosine reader protein YTH N6-methyladenosine RNA binding protein F1 (YTHDF1) promotes the development of MASH-HCC through the enhancer of zeste 2 polycomb repressive complex 2 subunit-interleukin 6 (EZH2-IL6) signaling pathway, as well as recruits and activates myeloid-derived suppressor cells. These effects lead to dysfunction of cytotoxic CD8^+^ T cells and induce immune tolerance in HCC [30]. Absence of PCK1 in MASH-HCC leads to the accumulation of 12-hydroxyeicosatetraenoic acid (12-HETE), which directly inhibits the effector function of CD8⁺ T cells, resulting in resistance to immunotherapy [31]. CD8^+^ T cells are the most important effector cells in immunotherapy.

Current research indicates that MASH can lead to an increase in CD8^+^ T cells in liver. Nevertheless, these cells are often located far from the tumor and may become functionally exhausted [25], leading to MASH-HCC immune tolerance [32]. Even in patients with MASH-HCC, CD8^+^ T cells exhibit a pro-cancer function, and the presence of CD8^+^ programmed cell death-1 (PD-1^+^) C-X-C motif chemokine receptor 6-positive (CXCR6^+^), thymocyte selection associated high mobility group box-positive (TOX^+^), and tumor necrosis factor-positive (TNF^+^) T cells increases the occurrence of HCC. This suggests that increased MASH-related CD8^+^ T cells have a pro-cancer effect rather than being involved in immune surveillance. Therefore, the role of T cells in patients with MASH-HCC requires re-evaluation [25]. Triggering receptor expressed on myeloid cells 2 (TREM2) acts on myeloid cells, promoting M2 polarization of macrophages and facilitating the release of neutrophil extracellular traps. Ultimately, this process leads to the differentiation of PD-1^+^ eomesodermin-positive (EOMES^+^) CD8^+^ T cells and regulatory T cells (Tregs), forming immune suppression. Knocking out TREM2 in myeloid cells can break the immune tolerance of MASH-HCC [33]. Cell cycle-related kinase collaborates with obesity-induced pro-inflammatory signals to promote the development of HCC associated with MASH. Cell cycle-related kinase induces co-occupancy and transcriptional upregulation of the STAT3-androgen receptor (STAT3-AR) promoter, forming a positive feedback loop. Subsequently, granulocyte colony stimulating factor (G-CSF) expression dependent on mechanistic target of rapamycin complex 1 (mTORC1) is activated through phosphorylation of glycogen synthase kinase 3 beta (GSK3β), thereby enhancing the recruitment and tumorigenicity of polymorphonuclear myeloid-derived suppressor cells and inducing immune tolerance in MASH-HCC [34].

Research indicates that there is widespread inactivation of mitochondrial carnitine palmitoyltransferase II (CPT II) in MASH [35]. Mitochondrial dysfunction caused by the inactivation of CPT II in T cells prevents the occurrence of effective immune responses in T cells [36]. Progression of MASH is often accompanied by an increase in neutrophil infiltration. Neutrophil extracellular traps formed by neutrophils can induce the differentiation of CD4 T cells into Tregs through toll like receptor 4 (TLR4), leading to the formation of an immunosuppressive microenvironment [37].

### MASH influences HCC immune tolerance via gut microbiota

**Fig. 1:**
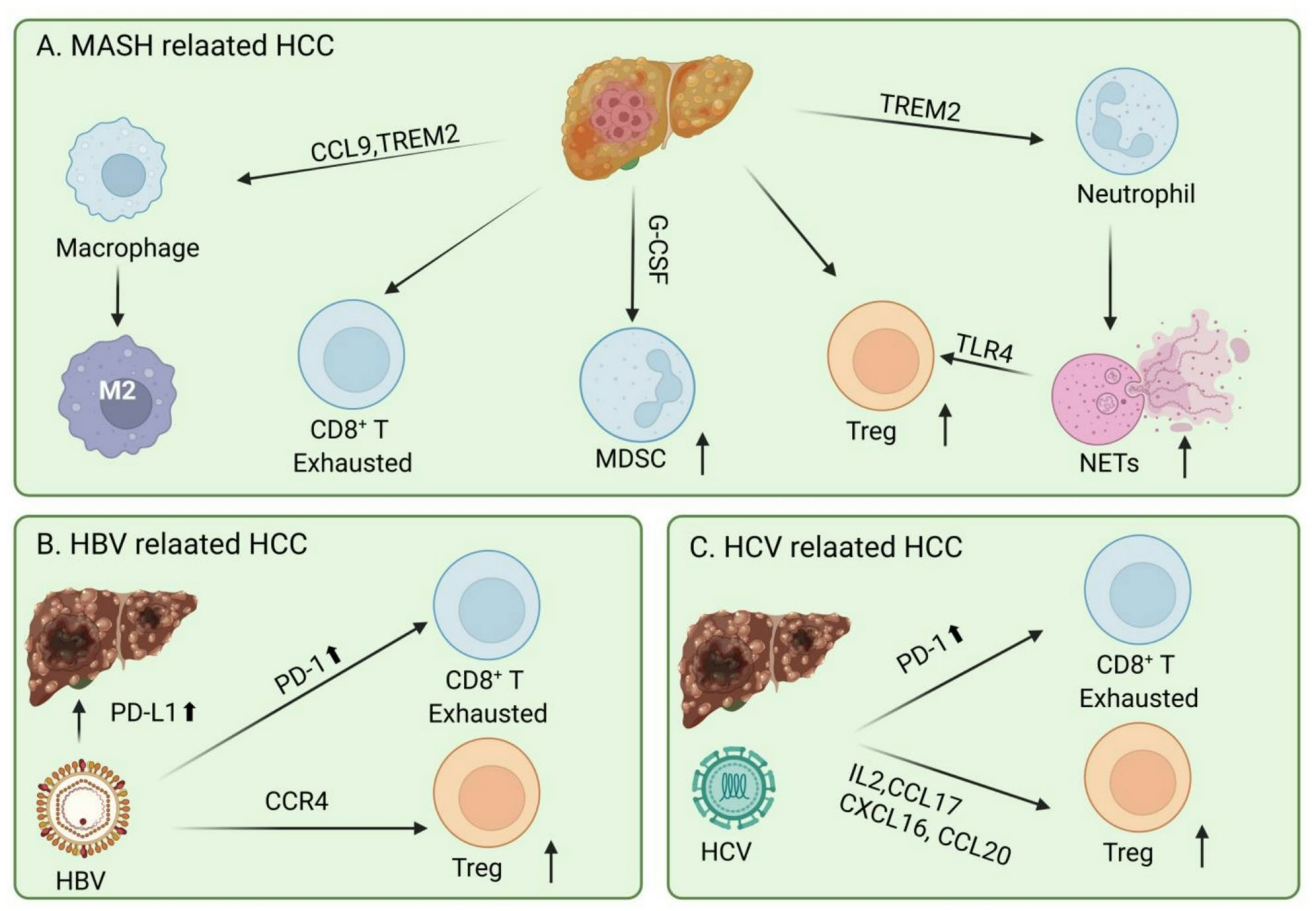
(**A**) Alterations in immune microenvironment in MASH-related HCC. (**B**) Alterations in immune microenvironment in HBV-related HCC. (**C**) Alterations in immune microenvironment in HCV-related HCC

The gut-liver axis plays a significant role in regulating tumor immune function [38]. It has been shown that certain bacterial species and short-chain fatty acids directly promote immunosuppressive responses [39]. Alterations in microbial composition, intestinal barrier damage, and dysbiosis are the primary mechanisms through which gut microbiota influence immune tolerance in MASH-HCC [40]. Patients with MASH-HCC exhibit elevated levels of certain short-chain fatty acids and their metabolic intermediates in their feces and serum. Moreover, their gut microbiota can induce immunosuppression, leading to the expansion of effector IL10^+^ Tregs in vitro, while simultaneously attenuating the expansion of cytotoxic CD8^+^ T cells [41]. Short-chain fatty acids produced by gut microorganisms increase Tregs and attenuate CD8^+^ T-cell responses, leading to immunosuppressive reactions [41]. Depletion of *Akkermansia mucilaginosus* was observed in MASH [42], and abundance of *Akkermansia mucilaginosus* bacteria is positively correlated with the response to PD-1 treatment. Thus, supplementing *Akkermansia mucilaginosus* in patients with MASH-HCC can restore their response to PD-1 [43] (Fig. 1A).

### Therapeutic targets for breaking MASH-HCC immune tolerance

Neutrophils expressing CXCR2 are enriched in MASH-HCC liver tissues. Inhibiting CXCR2 with AZD5069 can enhance the response to PD-1 in HCC patients with MASH [44]. Endocrine factor neuregulin 4 (NRG4) derived from adipocytes can block the formation of a MASH-induced immunosuppressive microenvironment [28]. Metformin can improve the mitochondrial activity and metabolism of MASH-HCC tumor-infiltrating CD8⁺ T cells, thereby exerting a PD-1-sensitizing effect and breaking down the immune tolerance of MASH-HCC [45]. N^6^-methyladenosine methyltransferase 3 (METTL3) promotes the development of nonalcoholic fatty liver disease-HCC. METTL3 inhibits antitumor immune responses by reducing the infiltration of granzyme B (GZMB⁺) and interferon-γ-positive (IFN-γ⁺) CD8⁺ T cells, thereby facilitating immune escape. METTL3 inhibitors (STM2457) or small-interfering RNA alone can inhibit MASH-HCC growth. Moreover, by enhancing CD8^+^ T cell cytotoxicity, these agents can synergize with PD-1 therapy to inhibit MASH-HCC progression [46].

## Role of HBV/HCV in immune tolerance of HCC

HBV infection is a major public health concern globally, affecting approximately 2 billion individuals, of whom > 350 million are chronic carriers [47]. The global infection rate of HCV is approximately 1.6%, affecting approximately 115 million individuals, of whom 71 million are in an active viremic state [48]. Although the incidence rate of HBV- and HCV-related hepatitis has significantly decreased in recent years, viral infections (HBV, HCV) remain the primary cause of HCC [49]. HBV infection causes continuous stimulation of hepatitis B surface antigen and hepatitis B e-antigen, leading to sustained activation and eventual exhaustion of CD8^+^ T cells. Sequencing data also indicate that HBV and HCV infections can form specific CD8^+^ T cells, which exhibit a state of functional exhaustion [50]. In response to inflammation caused by viral infection, the number of Tregs significantly increases in the liver infected with HBV, which also creates an immunosuppressive microenvironment [51]. Hepatitis B surface antigen achieves immune evasion by inducing T-cell exhaustion mediated by PD-1/PD-L1, stimulating the secretion of IL10/transforming growth factor-beta (IL10/TGF-β), and expanding the population of immunosuppressive myeloid-derived suppressor cells/Tregs [52]. In addition, HBV-induced secretion of inflammatory factors (TNF-α, IL6) is also a contributing factor to the exacerbation of immune tolerance [53]. As an RNA virus, HCV exerts a distinct impact on the liver microenvironment compared to HBV. HCV primarily undermines T-cell responses by influencing the antigen-presenting function of dendritic cells. Furthermore, HCV can also lead to an increase in the number of Tregs [54].

### Role of HBV infection in HCC immune tolerance

HBV is a DNA virus. Upon entering liver cells, it releases part of its double-stranded DNA genome, which is transported to the nucleus and converted into covalently closed circular DNA (cccDNA). The tenacity of cccDNA plays a central role in the persistent infection of chronic hepatitis B [55]. The cccDNA is also a key factor in the reactivation of HBV during immunotherapy, and the reactivated HBV can induce T-cell depletion in HBV-HCC [56]. During the progression of HBV infection, the innate immune system exhibits significant changes. Studies have confirmed that naked HBV-DNA can activate the cGAS/STING pathway to induce a strong innate immune response. However, this phenomenon has not been observed in HBV-infected hepatocytes. Various HBV infection models indicate that the HBV capsid can induce escape from the innate immune system, which is also an important contributor to HBV-HCC immune resistance [57, 58]. HBV can also accelerate the ubiquitin-dependent degradation of various pattern recognition receptors and their downstream adaptor proteins through the ubiquitin-proteasome pathway, leading to inhibition of the innate immune system [59]. HBV forms a ternary complex with hexokinase (HK) to sequester MAVS from RIG-I, thereby inhibiting the innate immune system [60]. In the treatment of HCC with PD-1, high levels of HBV DNA indicate poor patient prognosis and shortened overall survival. However, antiviral therapy can effectively improve the therapeutic effect of PD-1; hence, it may be crucial for patients with HBV-HCC [61]. HBV infection can lead to the upregulation of T-cell PD-1 and tumor cell PD-L1 in HCC. Therefore, combined use of PD-1/PD-L1 can significantly enhance the efficacy of immunotherapy and exhibit certain antiviral capabilities. This treatment holds excellent therapeutic prospects in HBV-HCC [62, 63]. The US Food and Drug administration has also approved the use of atezolizumab in combination with bevacizumab for the treatment of patients with HBV-HCC [64]. In HBV-HCC, long-chain acylcarnitine can induce the senescence of CD8^+^ T cells, and the unique lipid metabolism pattern of the liver is also an important inducer of HCC immune resistance [65].

In HBV-HCC, Kruppel-like factor 16 (KLF16) expression is significantly upregulated, while knockdown of KLF16 inhibits the growth and metastasis of HBV-infected HCC cells. HBV X protein-mediated N^6^-methyladenosine modification of KLF16 mRNA promotes the binding of insulin-like growth factor 2 mRNA-binding protein 2 (IGF2BP2) and IGF2BP3, thereby enhancing the stability of KLF16 mRNA. Further research has found that KLF16 promotes the transcription of chromosome 12 open reading frame 49 (C12orf49), which increases the expression of PD-L1 by competitively binding to speckle-type POZ proteins and blocking speckle type BTB/POZ protein-mediated (SPOP-mediated) PD-L1 ubiquitination and degradation. HBV promotes immune escape in HBV-HCC through the KLF16-C12orf49-PD-L1 axis. Importantly, inhibiting KLF16 significantly improves the efficacy of anti-PD-L1 treatment in HBV-HCC [66]. Currently, vaccines and specific chimeric antigen receptor T cells targeting HBV have also confirmed that anti-HBV can enhance the therapeutic effect of PD-1 [67]. As the primary type of Tregs in HBV-HCC, C-C motif chemokine receptor 4-positive (CCR4^+^) Tregs exhibit an immunosuppressive function, characterized by PD-1^+^ T cell factor-1-positive (TCF1^+^) stem cell-like properties. Blocking CCR4 can enhance the sensitivity of HCC to PD-1 [68] (Fig. 1B).

### Role of HCV infection in HCC immune tolerance

For patients with HCV-HCC, antiviral treatment should be administered with caution. While PD-1 treatment can suppress viral load, antiviral treatment may increase the recurrence rate in patients with HCV-HCC [69]. HCV infection also leads to the suppression of the innate immune system, primarily manifested by the upregulation of HCV-induced anti-interferon proteins. This suppression is mainly characterized by the blockade of interferon cascade signaling by the ubiquitin-proteasome pathway-related protein NS3/NS4A protease complex, the blockade of the NF-κB pathway by NS2 and NS5A, the inhibition of the STING–TBK1 pathway by NS4B, and the regulation of these proteins by Core Protein C, resulting in the suppression of the innate immune pathway [70]. HCV enhances the inhibitory phenotype and activity of Tregs, and increases Treg infiltration in the liver by inducing the expression of IL2, chemokines (CCL17, CXCL16, and CCL20), and chemokine receptors (CCR4, CXCR6, and CCR6) in Tregs, thereby inducing immune tolerance [71]. HCV induces CD8^+^ T-cell depletion by upregulating PD-1 expression and inhibiting IFN-γ production, ultimately promoting immune escape [72]. Relatively HCV-induced HCC exhibits a better response to immunotherapy [73] (Fig. 1C).

## Role of ALD in HCC immune tolerance

ALD and MASH share certain morphological similarities. Nonetheless, ALD (with inflammation as its core) further leads to metabolic changes which differ from those observed in MASH [56]. In addition, consumption of the carcinogen ethanol has led to increasing incidence and mortality rates among patients with ALD-HCC annually [61, 62]. Although the rate of ALD-HCC in patients with HCC is low, ALD represents a unique high-inflammatory immune microenvironment. In AIH-HCC, a large number of immune-related transcription factors exhibit temporal changes, being activated in the early stages of the tumor and suppressed in the later stages. Therefore, the immune resistance of HCC is also related to the progression stage of the tumor itself, and this issue deserves further attention [74]. Therefore, in this section, we specifically discuss the characteristics of immune tolerance in ALD-HCC.

Typical changes during the ALD process include increased intestinal permeability caused by ethanol, disruption of intestinal flora, and altered ethanol metabolism [63]. Long-term alcohol consumption can significantly upregulate the activities of alcohol dehydrogenase, aldehyde dehydrogenase, and cytochrome P450 2E1 (CYP2E1), leading to the generation of reactive oxygen species and activating the inflammatory response [64]. CCL20 is the most significantly upregulated cytokine during the ALD process [66]. In HCC, tumor-associated macrophages recruit Tregs through the CCL20-CCR6 axis, promoting HCC tolerance to PD-1 therapy [67].

## Role of therapeutic drugs for liver diseases on HCC immune tolerance

Liver diseases are common in patients with HCC. Treatment for liver diseases mainly includes lipid-lowering drugs, hepatoprotective drugs, antiviral drugs, and antifibrotic drugs. In addition, there are various novel targeted drugs for MASH. At present, the potential synergistic effects of these drugs on immunotherapy remain unknown. Research on the underlying mechanisms of such effects may help clinicians determine the most appropriate strategy for the management of primary disease during HCC treatment (Table 1).

**Table 1:** Mechanisms of liver disease-related drugs on HCC immune resistance

Drug Category	Drug / Agent	Proposed Mechanism in HCC Immune Tolerance	Evidence Status
Lipid-lowering	Statins (simvastatin)	Restore HSC quiescence via KLF2-NO; upregulate CXCL16 to recruit NKT cells; enhance anti-PD-L1 efficacy.	Clinical/Retrospective [78, 79]
	Fibrates	Inhibit PLTP to reduce M2 infiltration; disrupt PLTP-AURKA-P65 to suppress NF-κB.	Preclinical / In vitro [82]
Hepatoprotective	Glycyrrhizic acid (GA)	Inhibits NF-κB and mTOR pathways; potential liver-targeting nanocarrier.	Preclinical / In vitro [83, 84]
	Ursodeoxycholic acid (UDCA)	Improves CD8 + T cell function; synergizes with PD-1 therapy; induces HCC apoptosis.	Clinical / Animal models [96, 97]
Antiviral	Nucleotide analogs (NAs)	Suppresses HBV replication, reduces immunosuppressive microenvironment; improves ICI efficacy.	Clinical / Guideline-recommended [99–101]
	Interferon (IFN)	Enhances CD8 + T cell activity via metabolic remodeling; potential synergy with ICI but with toxicity.	Clinical / Phase II/III [104]
	Direct-acting antivirals (DAAs)	Effective against HCV; may reduce NK cell numbers, potentially contributing to immune tolerance.	Clinical / Observational [105–107]
Antifibrotic	Pirfenidone	Modulates DNA methylation/histone acetylation; regulates fibroblasts; inhibits Th2 differentiation.	Preclinical / Animal models [108–111]
	Nintedanib	Inhibits STAT3; regulates macrophage differentiation; direct antitumor effect.	Preclinical / In vitro [112, 113]
Other	GLP1 agonists (liraglutide)	Activates NK cell-mediated antitumor response via IL-6/STAT3 inhibition.	Preclinical / Animal models [114]

### Lipid-lowering drugs

Statins are commonly used cholesterol-lowering drugs, and previous studies have confirmed their effects on HCC prevention [75] and chemosensitization [76]. Simvastatin restores the quiescent state of activated hepatic stellate cells by stimulating the KLF2-nitric oxide (KLF2-NO) signaling pathway in hepatic sinusoidal endothelial cells, and upregulates the expression of CXCL16 in these cells, thereby recruiting natural killer (NK) T cells to inhibit tumor progression. Combined with anti-PD-L1 antibody treatment, targeted delivery of simvastatin has shown better therapeutic effects in mouse models [77]. These studies indicate that statins contribute to improving immune tolerance in HCC. However, a clinical study involving 295 participants revealed that atezolizumab-bevacizumab treatment for HCC was unaffected by MASH, suggesting that a combination therapy approach also circumvents immune tolerance in MASH-HCC [78]. Another clinical study involving 305 participants revealed that statins had no impact on the outcomes of HCC treated with the combination of atezolizumab and bevacizumab [79]. Therefore, the role of statins in HCC immunotherapy requires further interpretation through larger-scale preclinical or retrospective studies.

**Fig. 2:**
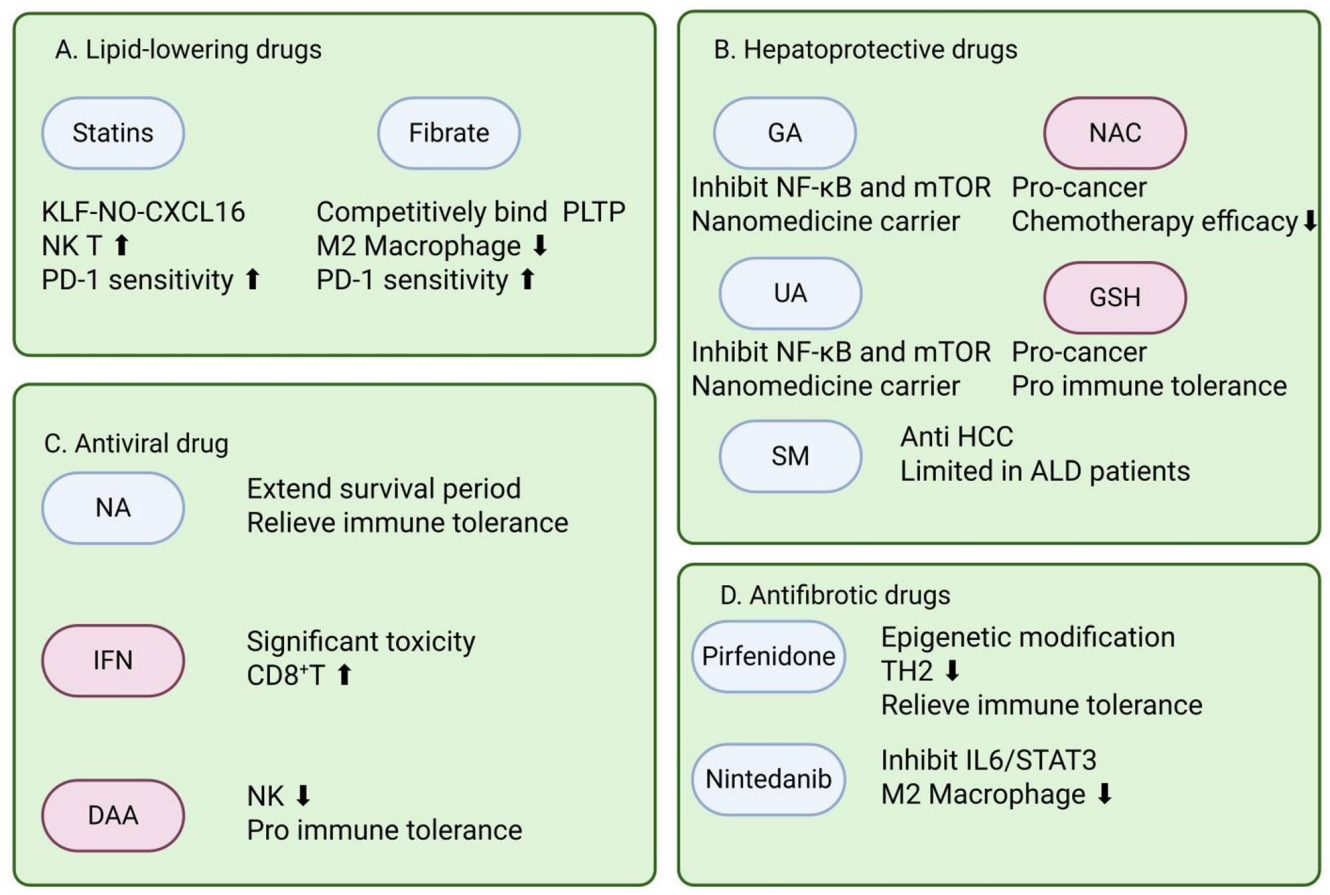
(**A**) Effect of lipid-lowering drugs on immune tolerance in HCC. (**B**) Effect of hepatoprotective drugs on immune tolerance in HCC. (**C**) Effect of antiviral drugs on immune tolerance in HCC. (**D**) Effect of antifibrotic drugs on immune tolerance in HCC

Fibrates, which are used to lower triglycerides, also exert inhibitory effects on HCC development [80] and enhance chemosensitivity [81]. They inhibit the upregulation of phospholipid transfer protein in HCC and bind to aurora kinase A (AURKA) and P65, forming a complex that induces P65 phosphorylation. This process activates nuclear factor-kappa B (NFKB) and upregulates the expression of IL6, IL8, and colony stimulating factor 1 (CSF1), promoting macrophage M2 polarization. Fibrates and GMB-475 can competitively bind to phospholipid transfer protein, thus inhibiting this process and increasing the sensitivity of HCC to PD-1 therapy [82]. However, further research is warranted to investigate the role of fibrates in immunotherapy for HCC(Fig. 2A).

### Hepatoprotective drugs

Glycyrrhizic acid (GA) is a commonly used anti-inflammatory and hepatoprotective drug. Previous studies have shown that GA has certain anti-HCC effects in vitro, exerting a certain degree of inhibition on the NFKB [83] and mTOR pathways [84], both of which play classic roles in HCC immune tolerance [85, 86]. In addition, GA is currently used as a nanomedicine carrier targeting the liver [87]. However, thus far, there is no direct evidence showing that GA can break the immune tolerance of HCC. This classic drug continues to hold profound potential in the treatment of HCC.

*Silybum marianum* is a classic natural antioxidant liver-protective drug that has demonstrated excellent performance in anticancer therapy. It also exhibits anticancer effects in HCC, including inhibition of the NOTCH pathway [88], regulation of the hepatocyte growth factor/c-Met (HGF/c-Met), Wnt/β-catenin, and PI3K/AKT/mTOR pathways [89], and enhancement of the effectiveness of chemotherapy for HCC [90]. Of note, *Silybum marianum* can accelerate the progression of ALD to HCC in male mice; therefore, it should be used with caution in patients with ALD [91].

N-acetylcysteine (NAC) is also an antioxidant hepatoprotective drug with dual effects in the treatment of HCC. It is also one of the hepatoprotective drugs not recommended for the treatment of patients with cancer. NAC can prevent the occurrence of HCC [92]. However, after the occurrence of HCC, NAC often has a pro-cancer effect and reduces the efficacy of chemotherapy [93]; nonetheless, latest research indicates that NAC may enhance the efficacy of immunotherapy [94]. However, the application of NAC in patients with HCC requires caution. Glutathione, another hepatoprotective drug with antioxidant function, has similar effects to NAC. While it can activate immune cells in the microenvironment, it promotes HCC progression and immune tolerance by inhibiting ferroptosis [95]. Therefore, antioxidant liver-protective drugs should be used with caution in patients with liver cancer. Ursodeoxycholic acid is another choleretic liver-protective drug that can improve the function of CD8^+^ T cells and enhance the therapeutic effect of PD-1 in HCC [96]. It can also directly induce HCC apoptosis [97] and serve as a drug carrier, exerting a better anti-HCC effect and offering promising application prospects [98] (Fig. 2B).

### Antiviral drugs

Nucleotide analogs (NA) are currently first-line anti-HBV drugs, and their use can effectively reduce the risk of HCC occurrence [99] and extend the survival period of patients with HBV-HCC [100]. HBV infection is one of the reasons responsible for the formation of an immunosuppressive microenvironment in HCC, and the use of NA to inhibit HBV can effectively improve the immune tolerance of HCC [101]. Interferon is also a commonly used drug for HBV; however, for patients with HBV-HCC, NA is a better choice. Although interferon has the potential to directly treat HCC [102], the drug is associated with significant toxicity [103]. Moreover, research studies reported that interferon can increase CD8⁺ T-cell activity [104]. Direct-acting antivirals are first-line therapeutic agents for HCV, which can effectively prevent the occurrence of HCC [105] and reduce the risk of HCC recurrence [106]. However, treatment with these agents can also reduce the number of NK cells in HCC, leading to immune tolerance [107] (Fig. 2C).

### Antifibrotic drugs

Pirfenidone is a classic antifibrotic drug used in the treatment of liver cirrhosis. It exerts anti-HCC effects by regulating DNA methylation and histone acetylation [108, 109]. In addition, it has the ability to regulate fibroblasts [110] and inhibit T helper 2 cell differentiation [111], which can improve the immune tolerance of HCC. However, these studies were limited to in vitro and animal experiments, and further clinical research is needed to confirm the findings. Nintedanib has been approved for the treatment of fibrotic lung diseases in idiopathic pulmonary fibrosis. At present, this drug is at the clinical research stage for use in the treatment of liver cirrhosis and HCC. Current research indicates that, in HCC, nintedanib can directly exert an antitumor effect by inhibiting STAT3 [112], and relieve immune tolerance by regulating macrophage differentiation [113] (Fig. 2D).

### Other new drugs

Glucagon-like peptide 1 (GLP1) agonists also demonstrate therapeutic potential in the management of MASH. Recent studies have shown that liraglutide can activate NK cell-mediated antitumor responses by inhibiting the IL6/STAT3 pathway in HCC, indicating that GLP1 agonists hold promising prospects for breaking immune tolerance in HCC [114].

Farnesoid X receptor agonists are commonly used in primary biliary cholangitis. Obeticholic acid can inhibit HCC progression by suppressing the IL6/STAT3 pathway [115], activate the antitumor effect of NK cells [116], and reduce the immune tolerance of HCC [117].

## Potential biomarkers of HCC immune resistance

In this section, we introduce molecules that have been clinically validated in current research and are associated with immune resistance in hepatocellular carcinoma (HCC), serving as potential biomarkers. High expression of Chromobox 4 inHCC and immunosuppressive tumor-associated macrophages (TAMs) subpopulations indicates non-responsiveness to PD-1 treatment [118]. MSR1^+^ TAMs induce CD8^+^ T cell depletion through the NF-κB/IL6 pathway, leading to non-responsiveness of HCC patients to PD-1 treatment [119]. High expression of Glyceronephosphate O-acyltransferase (GNPAT) predicts increased M2 infiltration in HCC, inducing immune resistance [120]. High expression of Hexokinase domain containing 1 (HKDC1) and USP14 impairs the metabolic function of CD8⁺ T cells in HCC, resulting in PD-1 resistance in HCC [121, 122]. High expression of Annexin A2 (ANXA2) is positively correlated with PD-L1 levels in HCC, leading to immune resistance [123]. High expression of the epigenetic regulator SETDB1 is positively correlated with the immunosuppressive microenvironment in HCC [124]. High expression of STE20/SPS1-related proline/alanine-rich kinase (SPAK) drives immune depletion in HCC and leads to resistance to PD-1 treatment [125]. High expression of Microspherule protein 1 (MCRS1) is positively correlated with immune resistance in HCC [126]. High expression of NQO1 increases Treg infiltration, leading to PD-1 treatment resistance in HCC [127]. High expression of Striatin Interacting Protein 2 (STRP2) is associated with reduced CD8⁺ T cell infiltration and immune resistance in HCC [128]. Increased infiltration of POSTN^+^ CAFs in HCC also indicates resistance to PD-1 treatment [129]. High expression of Galectin-4 induces immune resistance by regulating metabolic adaptability in HCC cells and recruiting neutrophils [130]. Transforming acidic coiled-coil-containing protein 3 and E3 ubiquitin ligase Riplet induce HCC immune resistance by regulating lipid metabolic reprogramming of CD8⁺ T cells [131, 132].

## Discussion and conclusions

Research indicates that the malignant progression of extrahepatic tumors is often accompanied by changes in liver function. Breast and pancreatic cancers induce a decrease in liver hepatocyte nuclear factor 4 alpha (HNF-4 A) during their development stages, leading to liver metabolic dysfunction and accelerating tumor progression [133]. This evidence also highlights the important role of liver function in tumor progression. When the liver is the primary tumor site, cancer often coexists with various primary liver diseases. However, current guidelines recommend the same treatment regimen for HCC in the context of different liver diseases [134]. T cell senescence and anergy are also important inducer of tumor immune resistance. However, research in this area is relatively scarce, and most of it has not been conducted in HCC with a clear disease context. Instead, experiments have been performed using CCL4 induction or orthotopic models. These studies indicate that IL-15 can inhibit CD8^+^ T cell senescence in HCC [135], while CD90^+^ eCAFs induce CD8^+^ T cell senescence through the secretion of LAMA4 [136]. We also look forward to validating these results in clinical samples and more specific models in the future. HCC cells compete with T cells for lysine uptake by overexpressing SLC3A2, leading to impaired STAT3 signaling, reduced proliferation, and decreased effector function of T cells [137]. Supplementation with lysine may enhance the efficacy of lenvatinib combined with PD-1 antibody. In HCC cells, LAIR1 upregulates PD-L1 expression by activating the GSK-3β/β-catenin/MYC signaling axis, thereby inducing a novel mechanism of CD8⁺ T cell depletion [138]. Targeted inhibition of LAIR1 helps restore the immune killing effect of T cells. Our review examined the differences in response to immunotherapy among HCC patients with different disease origins. There are numerous factors contributing to HCC immune tolerance. Spatial analysis based on multi-omics technology reveals that the spatial localization of CD8^+^ T cells is also a crucial determinant of immunotherapy response. The migration of CD8^+^ T cells to the tumor core area can influence the therapeutic efficacy of ICB. The alteration in the spatial localization of immune cells offers a novel explanation for the heterogeneity of immune tolerance in hepatocellular carcinoma [139]. In this article, we focus on the immune tolerance mechanisms of HCC originating from MASH, ALD, and HBV/HCV, including the regulation of the liver immune microenvironment and HCC by these primary liver diseases.

Immune resistance in different primary liver disease backgrounds exhibits certain commonalities and differences. Both MASH and HBV-induced immune resistance are accompanied by T cell exhaustion and upregulation of immunosuppressive receptors, yet the core mechanisms differ. In MASH-HCC, CD8^+^ T cell exhaustion and Treg increase are primarily caused by metabolic factors, including the accumulation of 12-HETE due to PCK1 deficiency, which directly impairs the effector function of CD8^+^ T cells [31], and mitochondrial dysfunction, such as CPT II inactivation, limits T-cell metabolic adaptability [35, 36]. In HBV-HCC, CD8^+^ T cell exhaustion is driven by persistent antigen and regulated by epigenetics, including the continuous presence of cccDNA providing uninterrupted antigenic stimulation. HBV-encoded proteins (such as HBx) stabilize PD-L1 expression through specific epigenetic axes, such as KLF16-C12orf49-PD-L1, via m6A modification [55, 66]. These differences suggest that reversing T cell exhaustion state in MASH-HCC may require metabolic reprogramming, while HBV-HCC may benefit more from the combination of antiviral therapy and epigenetic modifiers. Based on current research findings, for patients with HCC accompanied by MASH, it is advisable to utilize lipid-lowering and liver-protective drugs appropriately, as this can enhance the efficacy of immunotherapy. This also underscores the importance of long-term management of MASH. Patients with MASH often encounter the challenge of immune tolerance. Alternatively, more proactive approaches (e.g., selection of new targets) or treatment regimens (e.g., chimeric antigen receptor T cell therapy) may yield better therapeutic outcomes for patients with MASH-HCC [140]. For patients with HBV-HCC, it is reasonable to administer antiviral treatment, as antiviral drugs have synergistic effects with immunotherapy, facilitating the simultaneous treatment of the tumor and viral infection [141]. Additional clinical research results are needed to support the prioritization of immunotherapy use for patients with HBV-HCC in guidelines. However, the risk of viral rebound continues to pose a challenge. Moreover, the usefulness of prophylactic administration of antiviral drugs after immunotherapy remains unknown. The efficacy of antiviral treatment is greater for patients with HBV than for those with HCV; nonetheless, the response rate of patients with HCV to immunotherapy is significantly higher than that of patients with HBV. Nevertheless, these patients also face the risk of viral rebound [142].

The bile acid metabolism-gut microbiota axis is currently an important target for improving tumor immune tolerance. Various bile acids and gut bacteria that can improve immune tolerance have been identified in other tumors [143, 144]. Bile acid, as a drug carrier targeting the liver, also holds considerable potential for application in this field [145]. Currently, there are also some special types of HCC that deserve our attention. HCC cells deficient in ACVR2A produce and secrete lactic acid by upregulating the expression levels of lactate dehydrogenase A (LDHA) and monocarboxylate transporter 4 (MCT4). This effect promotes the accumulation of Tregs and subsequently confers resistance to immune checkpoint inhibitors. Knockdown of MCT4 can restore HCC sensitivity to PD-1 therapy [146]. The regulation of intestinal microbiota by different liver disease is not identical. MASH primarily leads to a decrease in the diversity of intestinal microbiota and an increase in the Enterococcus genus, resulting in the activation of NF-κB and STAT3 in the liver [147, 148]. The impact of HBV and HCV infections on the intestinal microbiota is mainly manifested as a reduction in beneficial bacteria (such as the Bifidobacterium genus), a decrease in short-chain fatty acid-producing bacteria like the Fecalibacterium genus, and an increase in pro-inflammatory bacteria like the Streptococcus genus [149]. ALD is represented by the damage to the microbiota and intestinal barrier caused by alcohol, often leading to translocation of intestinal microbiota [150]. These studies also indicate that different liver diseases have varying impacts on the remodeling of the intestinal microbiota. However, these results have not been further validated in hepatocellular carcinoma (HCC) with relevant disease backgrounds. Nevertheless, these findings suggest that different microbiota transplantation approaches should be adopted as precision treatment strategies for HCC with distinct disease backgrounds. With the continuous advancement of sequencing technology and the development of spatial omics, the individualization of immunotherapy for HCC in the future will help clinicians better address immune tolerance.

Our article still has certain limitations. Firstly, currently, there is only an animal model for MASH-HCC, and research on hepatocellular carcinoma induced by HBV, HCV, and ALD can only be conducted based on clinical data. However, most studies utilize subcutaneous tumor models or orthotopic models, which cannot support our viewpoint, and therefore, this part of the research has not been included in this article. Large-scale clinical cohort studies are still needed in the future to further explore this issue. Secondly, current research on hepatocellular carcinoma induced by liver diseases does not clearly stage the primary liver disease. The impact of the severity of liver disease on HCC immune tolerance remains unclear. We also anticipate that with the development of spatial omics, the development of organoid models, and the establishment of clinical cohorts in the future, these issues can be effectively addressed. In summary, the present article summarizes the differences in the tolerance of HCC to immunotherapy and the intervention pathways under various liver disease backgrounds. This review may provide a reference for the individualized treatment of HCC.

## Data Availability

No datasets were generated or analysed during the current study.

## Abbreviations

ALD: Alcoholic liver disease
GA: Glycyrrhizic acid
HBV: Hepatitis B virus
HCC: Hepatocellular carcinoma
HCV: Hepatitis C virus
MASH: Metabolic dysfunction-associated steatohepatitis
NA: Nucleotide analogs
NAC: N-acetylcysteine
NK: Natural killer
Treg: Regulatory T cell
